# Small Molecules for Multi-Wavelength Near-Infrared Fluorescent Mapping of Regional and Sentinel Lymph Nodes in Colorectal Cancer Staging

**DOI:** 10.3389/fonc.2020.586112

**Published:** 2020-12-17

**Authors:** Victor M. Baart, Marion M. Deken, Mark W. Bordo, Shadhvi S. Bhairosingh, Daniela C. F. Salvatori, Hoon Hyun, Maged Henary, Hak Soo Choi, Cornelis F. M. Sier, Peter J. K. Kuppen, Anton G. T. Terwisscha van Scheltinga, Taryn L. March, Adrianus R. P. M. Valentijn, John V. Frangioni, Alexander L. Vahrmeijer

**Affiliations:** ^1^Department of Surgery, Leiden University Medical Center, Leiden, Netherlands; ^2^Curadel, Natick, MA, United States; ^3^Central Laboratory Animal Facility, Leiden University Medical Center, Leiden, Netherlands; ^4^Anatomy and Physiology Division, Faculty of Veterinary Medicine, Utrecht University, Utrecht, Netherlands; ^5^Department of Biomedical Sciences, Chonnam National University Medical School, Gwanju, South Korea; ^6^Department of Chemistry, Center for Diagnostics and Therapeutics, Georgia State University, Atlanta, GA, United States; ^7^Gordon Center for Medical Imaging, Department of Radiology, Massachusetts General Hospital and Harvard Medical School, Boston, MA, United States; ^8^Department of Clinical Pharmacy and Toxicology, Leiden University Medical Center, Leiden, Netherlands

**Keywords:** image-guided surgery, fluorescence, cancer staging, ZW800, indocyanine green

## Abstract

Assessing lymph node (LN) status during tumor resection is fundamental for the staging of colorectal cancer. Current guidelines require a minimum of 12 LNs to be harvested during resection and ultra-staging regional lymph nodes by sentinel lymph node (SLN) assessment is being extensively investigated. The current study presents novel near-infrared (NIR) fluorescent dyes for simultaneous pan lymph node (PanLN; regional) and SLN mapping. PanLN-Forte was intravenously injected in mice and assessed for accumulation in regional LNs. SLN800 was injected intradermally in mice, after which the collection and retention of fluorescence in SLNs were measured using indocyanine green (ICG) and its precursor, SLN700, as references. LNs in the cervical, inguinal, jejunal, iliac, and thoracic basins could clearly be distinguished after a low dose intravenous injection of PanLN-Forte. Background fluorescence was significantly lower compared to the parent compound ZW800-3A (p < 0.001). SLN700 and SLN800 specifically targeted SLNs with fluorescence being retained over 40-fold longer than the current clinically used agent ICG. Using SLN700 and SLN800, absolute fluorescence in SLN was at least 10 times higher than ICG in second-tier nodes, even at 1 hour post-injection. Histologically, the fluorescent signal localized in the LN medulla (PanLN-Forte) or sinus entry (SLN700/SLN800). PanLN-Forte and SLN800 appear to be optimal for real-time NIR fluorescence imaging of regional and SLNs, respectively.

## Introduction

Adequate yield and correct assessment of lymph node (LN) involvement in colorectal cancer (CRC) is of fundamental importance for determining the prognosis of cancer patients and guiding treatment decisions (1). Therefore, current guidelines of the National Comprehensive Cancer Network and European Society for Medical Oncology endorse a minimal harvest of 12 LNs as quality indicator for CRC resections (1, 2). Obtaining an acceptable LN yield remains an important issue, especially in left-sided and smaller tumors, older patients and after neoadjuvant treatment where the minimal harvest is reached less often (3). In addition to gross lymph node examination, ultra-staging regional nodes by a sentinel lymph node (SLN) procedure is being investigated for refining staging and subsequent adjuvant therapy assignment (4). However, the sensitivity, ranging from 0.33 to 1.00, remains an issue (4, 5). Both regional LN (all lymph nodes; referred to as PanLN henceforth) harvesting and SLN procedures could benefit from improved detection techniques.

Ionizing as well as non-ionizing agents based on near-infrared (NIR) light have been used to identified (S)LNs. The NIR I and II windows are advantageous due to the favorable penetration depth (up to 5–8 mm), low tissue absorption and scattering, and minimal tissue autofluorescence (6). Consequently, the use of indocyanine green (ICG, a 800 nm NIR I fluorescent dye) for SLN-detection has been studied extensively in patients with malignancies of breast, skin, vulva, bladder, prostate, cervix, endometrium, ovarium, esophagus, stomach and colon (7–11). Unfortunately, ICG passes rapidly through the SLN towards deeper LNs, leading to unnecessary removal of these nodes (12). Another approach utilizes quantum dots to target SLNs as they have a high quantum yield (QY) and narrow bandwidth, permitting simultaneous visualization of up to five separate SLN dyes (13). However, their clinical translation is currently hampered due to their semiconductor-containing cores (13–16). In contrast to SLN tracers, research into panLN tracers is sparse. One approach using Cy5.5 polymers suffered from a high non-specific background signal after intravenous injection (17). By engineering novel small molecule-based polymethine cyanine fluorophores we recently visualized SLN as well as PanLNs at two distinct fluorescence emissions: 700 nm for the SLN agent and 800 nm for the PanLN agent, in small and large animal models (18). These dyes have meanwhile been refined with respect to their optical properties and pharmacokinetics.

The objective of the current study was to evaluate derivatized SLN and PanLN fluorophores for the detection of regional and sentinel LNs by *in vivo* NIR fluorescence imaging in mice. PanLN and SLN targeting efficiencies were measured in real-time using mice models, and post-mortem histological analyses were used to verify lymphatic uptake.

## Materials and Methods

### Syntheses of Contrast Agents

Polymethine contrast agents generally have similar synthetic pathways that include a condensation reaction to combine two side groups with a central core. These side groups are usually functionalized indoles, whereas the central core contains the unsaturated hydrocarbon chain. In the case of the PanLN agents, the central core of the first NIR intermediate contains a halogen group that is used in a substitution reaction to add additional chemical groups to the molecule.

**Figure 1:**
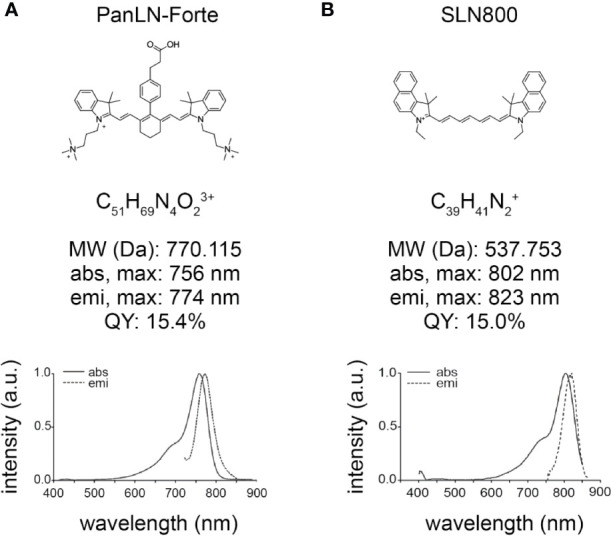
Chemical structure and optical properties of 800 nm lymph node fluorophores in HEPES-buffered serum, pH 7.4. **(A)** PanLN-Forte and **(B)** SLN800. abs, absorption; a.u., arbitrary units; Da, Dalton; emi, emission; max, maximum; MW, molecular weight; nm, nanometer; QY, quantum yield.

The reference compound ZW800-3A (19) and the novel compound PanLN-Forte (**Figure 1A**) syntheses start with the alkylation of commercially available 2,3,3-trimethyl-3H-indole with the salt (3-bromopropyl)trimethylammonium bromide. This indole is then used in a 2:1 ratio during a condensation reaction to produce the first NIR intermediate. The halogenated NIR intermediate is then used in an SN1 reaction with SOPP to form ZW800-3A, or used in a Suzuki-Miyaura coupling to form PanLN-Forte.

The SLN contrast agent’s indoles are prepared with the alkylation of the commercially available 1,1,2-trimethylbenz[e]indole with iodoethane. The functionalized indole is then reacted with either a 3-carbon or 5-carbon central core to produce the reference compound SLN700 (18) and the novel compound SLN800 (**Figure 1B**), respectively.

All polymethine contrast agents were synthesized from chemicals and solvents that were of ACS grade or greater. Starting materials were purchased from Sigma-Aldrich (Saint Louis, MO) or Fisher Scientific Inc. (Pittsburgh, PA) and used without purification.

Final products were either purified by a series of solvent washes or by solid phase extraction using PoraPak Rxn sorbents (Waters, Milford, MA). Chemical identification and analysis of the contrast agents were completed on an Acquity UPLC-MS (Waters, Milford, MA).

### Optical Property Analysis

Optical properties were measured in fetal bovine serum (FBS) supplemented with 100 mM HEPES, pH 7.4 or phosphate-buffered saline (PBS), pH 7.4. Quantum yields (QY) were determined in dimethyl sulfoxide with the comparative method using ICG (QY 13%) as calibration standard. Optical measurements were collected on a Cary 50 Bio UV-Visible spectrophotometer, Cary Eclipse fluorescence spectrometer, respectively (Varian/Agilent, Mattapoisett, MA).

### Animal Models

Animal experiments were approved by the Dutch Central Commission for Animal experimentation (Centrale Commissie voor Dierproeven). Mouse experiments were performed at the Central Animal Facility of the Leiden University Medical Center (LUMC). All animals were SPF as commended by the FELASA recommendation (20). Six-month-old female NMRI-mice (Charles River laboratories, l’Arbresle, France) were used for all experiments except for studies evaluating fluorescence retention in the SLN, where 6–10 week-old Foxn1<nu> mice (Charles River laboratories, l’Arbresle, France) were used. Each test condition consisted of three to five mice.

### NIR *In Vivo* Imaging Systems

The Pearl Trilogy Small Animal Imaging System (LI-COR Biosciences, NE, USA) allows white-light and near-infrared imaging at 700 and 800 nm in a black-box setting, minimizing the interference from ambient light. It is used as a gold standard for quantification and comparison of *in vivo* fluorescent signals. To demonstrate the potential of the dyes for clinical translation the mini-FLARE Imaging Systems Kit, FLARE Model R1 (both Curadel, Natick, MA, USA) and Artemis (Quest Medical Imaging, Netherlands) near-infrared camera systems were used. Each of these systems is equipped with 700 nm and 800 nm channels next to visual color, and is suitable for intraoperative use. Using mini-FLARE, excitation wavelengths for 700 nm and 800 nm NIR fluorophores were 665 ± 1 nm and 760 ± 1 nm, respectively, with typical fluence rates of 1–10 mW/cm2.

### *In Vivo* LN Imaging

PanLN-Forte and ZW800-3A (Curadel, Natick, MA, USA) were administered intravenously at the doses indicated in the tail vein of NMRI-mice. Four hours after injection the sub-iliac, proper axillary, accessory axillary, mandibular, accessory mandibular, superficial parotid, jejunal, medial iliac, external iliac, tracheobronchial and caudal mediastinal LNs were identified as described by Van de Broeck et al., followed by imaging and resection (21). Previous work demonstrated that 4 hrs post-injection is the optimal imaging moment for PanLNs and 5 nmol is the optimal dose for ZW800-3A, which served as reference (18). Accordingly, concentrations of 1, 5, and 10 nmol PanLN-Forte in 100 µl phosphate buffered saline (PBS) were tested. Biodistribution of the PanLN agents was determined at 5 minutes (min) and 4 hrs post-injection.

Ten microliters of 30, 125, and 500 µM SLN700 and SLN800 dissolved in 50% ethanol:PBS were administered intradermally in the footpad of NMRI-mice. SLNs were dissected, imaged, resected, and confirmed by histology (described below). Fluorescence retention in the SLN was assessed by intradermal injection of 10 µl dye at the left side of the base of the tail in Nu/Nu mice. Lymph flow, first to the left sub-iliac LN (first tier node, SLN) and subsequently to the left axillary LNs (second tier node, distal LNs) was followed for a maximum of 15 min. Imaging was repeated at 30 min and 60 min, respectively, and after 24 hrs. All mice were kept under isoflurane anesthesia during injection and imaging. Based on previous studies, 500 µM ICG was used as a control (22).

### Histological Analysis

Resected tissue specimens were fixed overnight in 4% formalin, embedded in paraffin, sectioned (5 µm), and scanned with the Odyssey Clx Infrared Imaging System for NIR-fluorescence (LI-COR Biosciences, NE, USA). Sections were subsequently stained with hematoxylin-eosin, digitalized with the Pannoramic Digital Slide Scanner, and viewed with CaseViewer 2.3 (both 3D Histech, Hungary). A European certified veterinary pathologist (ECVP) confirmed the presence of lymphoid tissue. Merged images were generated for ultrastructural evaluation of the fluorescent location.

### Quantification and Statistical Analysis

Pearl and Odyssey Clx images were analyzed with Image Studio Ver5.2 (LI-COR Biosciences, NE, USA). FLARE images were analyzed using FLARE-software (Curadel, Natick, MA, USA). Artemis images were captured with Spectrum Capture Suite 1.4.3 (Quest Medical Imaging, Netherlands) and analyzed with Fiji Image-J (23, 24). Signal-to-background ratios (SBRs) were measured by drawing a region of interest (ROI) around the LN of interest and a second ROI of similar size on the surrounding fat or muscle when no fat was present, and subsequent division of both mean fluorescent intensities (MFI). Results are reported as mean with standard deviation (SD). Means were compared using t-tests with IBM SPSS Statistics 23 (IBM Nederland BV, The Netherlands). Only p-values equal to or below 0.05 indicated significance.

## Results

### Optical and Chemical Properties

PanLN-Forte has strong optical properties in serum with a maximum absorbance wavelength of 756 nm and emission wavelength of 774 nm (**Figure 1A**). The molar extension coefficient at 756 nm is 158,300 M^−1^·cm^−1^ and the QY is 15.4%. SLN800 has a similar molar extinction coefficient at 156,300 M^−1^·cm^−1^, but with higher peak absorbance/emission wavelengths, and a QY of 15.0%. The maximum absorbance and emission wavelengths for SLN800 are 802 and 823 nm, respectively. SLN700 has a molar extinction coefficient of 157,400 M^−1^·cm^−1^, a maximum absorbance at 692 nm, and a maximum emission wavelength of 714 nm. Mass Spectrometry show M+3 for PanLN-Forte and M+ for SLN800 (**Supplementary Figure 1**).

### PanLN Imaging

**Figure 2:**
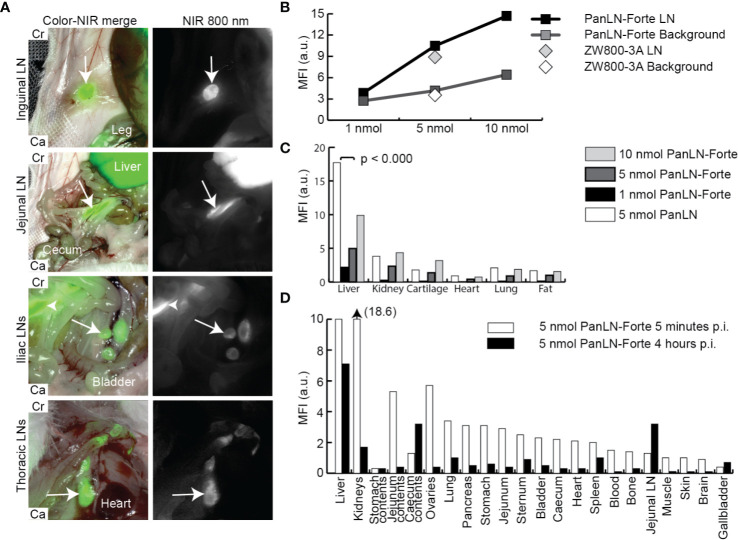
Regional lymph node imaging with PanLN-Forte at > 780 nm emission. **(A)** Important LNs located in superficial fat (inguinal LN), the abdomen (jejunal and iliac LNs), and thorax could be distinguished from the background 4 hrs after injection of PanLN-Forte. Arrows indicate the respective LN while the arrowhead points to the jejunal LNs. Images taken with the FLARE Model R1. **(B)** MFI of signal and background for ZW800-3a and PanLN-Forte. **(C)** Dose-dependent uptake ZW800-3a vs. PanLN-Forte. **(D)** Biodistribution at 5 min and 4 hrs after injection of PanLN. a.u., arbitrary unit; Ca, caudal; Cr, cranial; LN, lymph node; MFI, mean fluorescent intensity; NIR, near-infrared; nm, nanometer; nmol, nanomole; p.i., post injection.

PanLN-Forte specifically accumulated in clinically important superficial, abdominal and thoracic LNs, and the MFI increased with dose (**Figures 2A, B**, **Supplementary Video 1**). With all three dose groups and NIR imaging systems SBRs were > 2.0 except for the 1 nmol dose group (**Supplementary Table 1**). While the MFI values of the LNs after PanLN-Forte injection were similar to those of the parent compound ZW800-3A (p = 0.753, **Figure 2B**), the non-specific signal in the liver was significantly reduced (p < 0.001, **Figure 2C**). Biodistribution analysis revealed that PanLN-Forte was excreted into the bile within 5 min after injection. Non-specific fluorescence had cleared sufficiently after 4 hrs to allow jejunal LN visualization as the MFI of the jejunal LNs was > 2 higher than all organs except for the liver (**Figure 2D**).

### SLN Imaging

**Figure 3:**
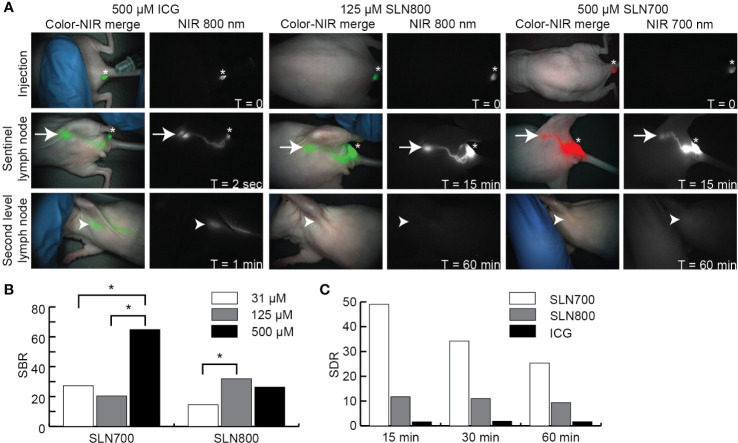
Near-infrared sentinel lymph node imaging. **(A)** NIR fluorescent images illustrating over time (0–60 min) the fluorescence at the injection site, SLN and second tier node (distal node) after intra-dermal ICG, SLN800, and SLN700 injection. Asterisks indicate injection sites, arrows the SLN and arrowheads the location of second tier nodes. Images taken with the Artemis excited with a 785 laser for ICG and SLN800 and 680 nm laser for SLN700. **(B)** SBRs of various doses SLN700 and SLN800. **(C)** SLN-to-distal node ratio (SDR) of SLN700, SLN800, and ICG over time (0–60 min). ICG, indocyanine green; SLN, sentinel lymph node; µM, micromolar; min, minutes; NIR, near-infrared; nm, nanometer; SBR, signal-to-background ratio; SDR, SLN-to-distal node ratio; sec, seconds; T, times.

SLNs could be visualized at a lower dose of SLN800 than the clinically used ICG dose (125 µM vs 500 µM, **Figure 3A**). SBRs for 500 µM and 125 µM SLN800 were similar and differed significantly from 31 µM SLN800 (125 µM vs. 31 µM: p = 0.008, **Figure 3B**). The optimal dose of SLN800 was set at 125 µM, as it was the lowest dose that still resulted in the requisite SBR of > 2 with all three fluorescent imaging systems. For SLN700 the optimal dose was set at 500 µM, as this gave the highest SBRs (500 µM vs. 125 µM: p = 0.001; 500 µM vs. 31 µM: p = 0.008, **Figure 3B**). While ICG rapidly passed to distal nodes (non-SLN), SLNs retained SLN700 and SLN800 over 40 times longer (14 ± 3 seconds vs. 17 ± 9 min vs. 10 ± 7 min, respectively, **Figure 3A**). Using SLN700 and SLN800, absolute fluorescence in the SLN was at least ten times higher than that in second-tier nodes, even at 1 hr post-injection (**Figure 3C**). This allowed for discrimination of SLNs from second-tier nodes using MFI long after the fluorescence has passed the SLN with SLN700 and SLN800.

### Ultrastructural Fluorescence of LN Dyes

**Figure 4:**
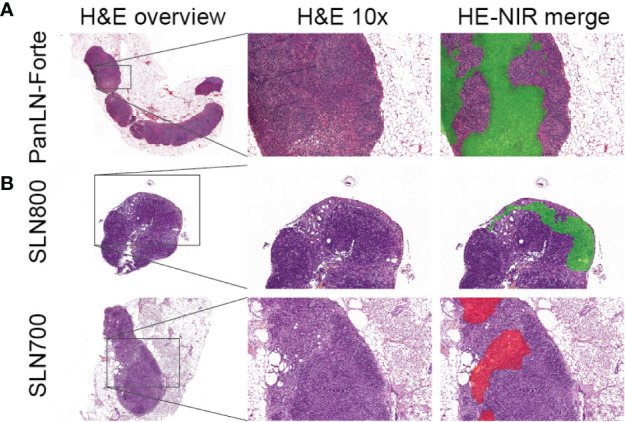
Ultrastructural analysis of lymph node fluorescence. **(A)** PanLN-Forte. 800 nm fluorescence (green pseudo-color) was seen throughout the medulla. **(B)** SLN800 and SLN700. 800 nm fluorescence (green pseudo-color) and 700 nm fluorescence (red pseudo-color) localized to the subcapsular sinus after intra-dermal injection. An overview H&E staining and 10x magnification are shown, after which the 10x H&E was merged with the fluorescence images. H&E, hematoxylin & eosin; NIR, near-infrared.

Intravenous injection of PanLN-Forte identified 94 LNs in 13 mice. All LNs in the 5 nmol dose group were resected for histology and contained lymphoid tissue. Histologically, the fluorescence localized towards the medulla of the LN (**Figure 4A**).

Twenty-nine of 31 SLNs identified with SLN800 were histologically confirmed as lymph nodes. Although visually confirmed during resection, 2 SLNs could not be histologically verified due to loss or damage of tissue during surgical resection and subsequent histological preparation. All 19 SLNs found with SLN700 were histologically confirmed. Fluorescence accumulating in the subcapsular sinus confirmed lymphatic sinus entry of the dye (**Figure 4B**).

## Discussion

In CRC, resecting a minimal of 12 LNs has been related to prognosis and survival outcomes and therefore been incorporated in various international guidelines (1–3). Consequently, the proportion of surgeries in which 12 or more LNs have been resected has risen from 13%–35% at the turn of the century to 59%–74% (25–28). While the majority of LN metastasis in CRC are found in nodes smaller than five millimeter, the current techniques of visualization and palpation bias’s towards identifying larger LNs (29). Current navigation techniques do not identify PanLNs. Furthermore, SLN-biopsy investigations are hampered by the currently available SLN-agents as they are neither real-time, retained in the SLN, aesthetically pleasing due to tattooing, nor conserving of the surgical field.

The current *in vivo* study describes improved PanLN and SLN imaging agents ready for clinical translation. After intravenous injection, PanLN-Forte exhibited reduced background signal in key organs such as the liver, unlike the previously described Cy5.5 polymers and ZW800-3A, while maintaining PanLN homing ability (17, 18). Despite finding uptake of PanLN-Forte in the liver and, to a lesser extent, in cartilage, these structures can be shielded during a surgical procedure, and are generally far away from the mesenteric LNs that play a relevant role in CRC LN metastasis (30). The optimal dose of 5 nmol translates to a human equivalent dose of 0.015 mg/kg, which is a low dose and similar to that of other recently clinically tested fluorophores for anatomic enhancement (31, 32). Histologically, PanLN-Forte accumulated in the medulla of LNs suggesting direct uptake from the systemic circulation. Although the exact mechanism of uptake is not currently known, phagocytic cells, such as alveolar macrophages and dendritic cells, preferentially engulf cationic structures (33).

SLN800 identified SLNs in real-time and with a substantially lower dose than ICG (125 µM vs. 500 µM). Partially, this can be attributed to the higher QY of SLN800 than ICG (15% vs. 9%) (34). Furthermore, SLN800 and SLN700 were retained in the SLN over 40 times longer than ICG. The improved performance of the SLN dyes versus ICG is not understood completely. Typically, an ideal agent for SLN targeting is large (≥ 10–50 nm hydrodynamic diameter) and has high charge, high hydrophobicity, or both (35, 36). Hence, the rapid clearance of ICG out of the lymphatics can be attributed to its small hydrodynamic diameter of 1.2 nm and amphiphilic structure. Previously, ICG has been mixed with human serum albumin (HSA) to increase its size to 7.3 nm, but in clinical trials, ICG:HSA did not result in improved SLN detection over ICG alone (37). The chemical structures of SLN700 and SLN800 offer clues as to why they have such high retention compared to ICG. SLN800 and SLN700 are both much more hydrophobic than ICG and have a cationic charge from the indole nitrogen. This combination of charge and hydrophobicity either increases effective hydrodynamic diameter by binding to proteins in lymph, or is a trigger for uptake by phagocytic lymphatic cells at the sinus entry point. Either way, the net effect is higher retention in the SLN.

As the NIR fluorescence imaging field evolves, and as AI-directed robotic surgery commences, dual-wavelength (i.e., multiplexed) navigation will become increasingly important. With the introduction of two-channel NIR-camera systems operating with excitations at 700 nm as well as 800 nm, surgeons and/or robots can focus on two targets at the same time. The combined optical properties of PanLN-Forte and SLN700 permit simultaneous visualization of both the SLN and all other nodes in a tumor basin. Furthermore, in colorectal cancer the combination of PanLN-Forte with SGM-101, a 700 nm CEA-targeting tumor-specific agent currently in phase III trials (NCT03659448), would facilitate the identification of both the tumor and the LNs (38). This is especially relevant in rectal cancer patients treated with neoadjuvant (chemo) radiotherapy where extensive fibrosis and scar tissue make tumor and LN identification difficult (39). SLN700 and SLN800 also permit concurrent SLN and tumor imaging with the whole arsenal of tracers in various study protocols, and future development may permit the use of dual-wavelength NIR fluorescence to both find the SLN and identify micro-metastases in real-time (40). Finally, the potential of SLN700, SLN800 and PanLN-Forte is not only limited to colorectal cancer but also applicable to other tumor types like non-small cell lung cancer where a minimum of 10 LNs need to be resected and vulvar, head-and-neck and esophageal cancer where SLN-biopsies are being studied extensively (7, 41).

A possible limitation of the current study is that animal experiments were not performed in tumor-bearing mice, which could have altered lymph drainage. However, previous studies suggest no difference in tumor-bearing and non-tumor-bearing animals with respect to SLN identification (42). Another limitation might arise during clinical translation, where high BMI, previous neoadjuvant therapy, or previous abdominal procedures could hamper LN visualization. In fact, visual enhancement methods are most needed in these clinical settings (43).

The novel 800 nm contrast agents PanLN-Forte and SLN800 appear to be optimal for real-time NIR fluorescence imaging of regional and sentinel lymph nodes, respectively, may be used in conjugation with 700 nm contrast agents for multi-wavelength surgical guidance, and rapid clinical translation is expected.

## Data Availability Statement

The raw data supporting the conclusions of this article will be made available by the authors upon reasonable request.

## Ethics Statement

The Dutch Central Commission for Animal Experimentation approved all animal experiments (AVD1160020172925). Experiments were performed in accordance with the code of practice “Dierproeven In Het Kankeronderzoek”.

## Author Contributions

The manuscript has been seen and approved by all authors. VB, MD, and SB executed the preclinical studies. DS trained the preclinical researchers and analyzed the histological data. MB, HH, MH, and HC developed the dyes. MB, AT, TM, and AV were responsible for the production of the dyes and analysis of chemical data. CS, PK, JF, and AV coordinated and supervised the experiments. VB wrote the manuscript with assistance from all the contributing authors. All authors contributed to the article and approved the submitted version.

## Funding

Research reported in this study was supported in part by the National Cancer Institute (NCI) of the National Institutes of Health under award number R01-CA-207500. The content is solely the responsibility of the authors and does not necessarily represent the official views of the National Institutes of Health. CS was in part funded by the European Commission under two Marie Skłodowska-Curie Action awards: H2020-MSCA-RISE-2019 (Project number: 872860 - PRISAR2) and H2020-MSCA-ITN-2019 (Project number: 857894 - CAST).

## Conflict of Interest

Author JF is founder and CEO of Curadel, LLC. Author MB was employed by the company Curadel, LLC.

The remaining authors declare that the research was conducted in the absence of any commercial or financial relationships that could be construed as a potential conflict of interest.

## Supplementary Material

Supplementary Video 1 NIR identification and resection of LNs after injection of 5 nmol PanLN-Forte. Fluorescence imaging was done using the Artemis (Quest, Netherlands). LNs were confirmed by pathology.
