# Supplementary material for: Small Molecules for Multi-Wavelength Near-Infrared Fluorescent Mapping of Regional and Sentinel Lymph Nodes in Colorectal Cancer Staging
Source: Front Oncol. 2020 Dec 17;10:586112. doi: 10.3389/fonc.2020.586112 (PMC7774022; doi:10.3389/fonc.2020.586112)
Supplement: Supplementary file 2 [file DataSheet_1.docx]

Supplementary Material

# Supplementary Figures and Tables

## Supplementary Figures

**
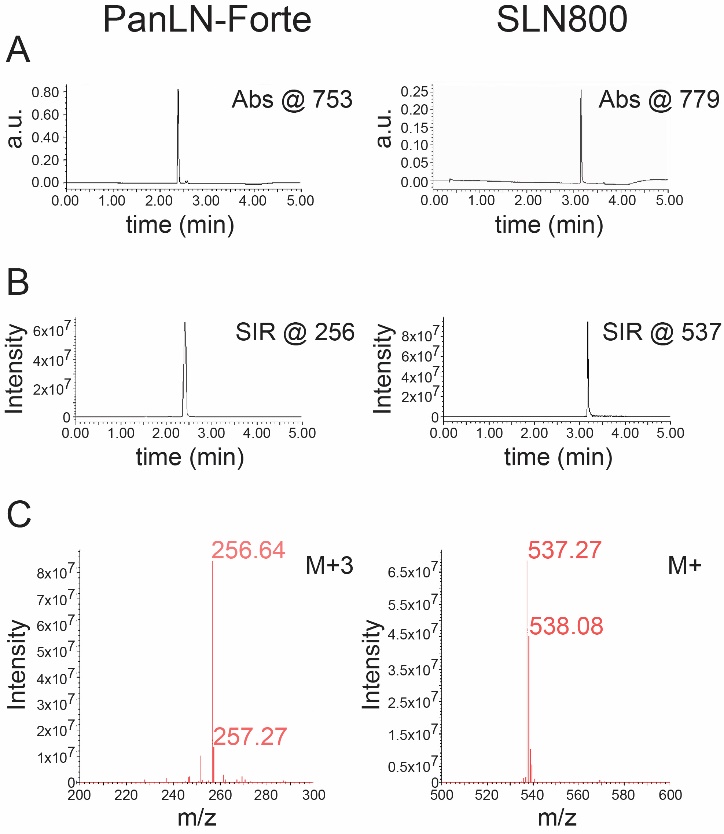
**

**Supplementary Figure 1.** UPLC-MS analysis of PanLN-Forte (left column) and SLN800 (right column). (A) NIR absorption chromatogram (B) SIR chromatogram (C) MS of PanLN-Forte (m/z = 256) and SLN800 (m/z = 537). a.u., arbitrary unit; min, minutes; m/z, mass-to-charge ratio; NIR, near-infrared; SIR, selected ion recording; UPLC-MS, ultra-performance liquid chromatography – mass spectrometry.

## Supplementary Tables

**Supplementary Table 1.** SBRs of near-infrared lymph node dyes with three different imaging platforms 4 hours after injection in mice models for PanLN-Forte and directly after injection of SLN800 and SLN700.

| Tracer | Dose | NIR-imaging systems | | |
| --- | --- | --- | --- | --- |
|  |  | Pearl SBR (SD) | Flare SBR (SD) | Artemis SBR (SD) |
| PanLN-Forte | 10 nmol | 3.9 (0.3) | 2.4 (0.3) | 2.3 (0.7) |
|  | 5 nmol | 4.0 (0.7) | 2.7 (0.9) | 2.1 (0.2) |
|  | 1 nmol | 4.5 (0.5) | 1.5 (0.1) | 2.5 (0.9) |
| SLN800 | 500 µM | 26.3 (8.6) | 7.0 (1.8) | 10.5 (1.9) |
|  | 125 µM | 31.9 (8.5) | 7.6 (5.3) | 11.1 (2.9) |
|  | 31 µM | 14.5 (9.8) | 1.6 (0.3) | 6.3 (3.6) |
| SLN700 | 500 µM | 64.9 (15.8) | 7.8 (1.7) | - |
|  | 125 µM | 20.5 (12.0) | 3.0 (1.7) | - |
|  | 31 µM | 27.2 (24.7) | 4.2 (4.8) | - |

NIR, near-infrared; SBR, signal-to-background ratio; SD, standard deviation.
